# An economic evaluation of breast cancer interventions in Kenya

**DOI:** 10.1016/j.eclinm.2024.102894

**Published:** 2024-10-30

**Authors:** Brian Hutchinson, Rory Watts, Mary Nyangasi, Benjamin O. Anderson, Joyfrida Chepchumba, Elizabeth Wangia, Rose Jalang'o, Valerian Mwenda, Pooja Yerramilli, Toni Lee Kuguru, Kenneth Munge Kabubei, Amparo Gordillo-Tobar, Filip Meheus, Christina Meyer, Andre Ilbawi, Rachel Nugent

**Affiliations:** a Center for Global Noncommunicable Diseases, International Development Group, RTI International, 3040 East Cornwallis Road, P.O. Box 12194, Research Triangle Park, NC, 27709-2194, USA; b Forecast Health, 38 Queen Victoria Street, Fremantle, Western Australia, 6160, Australia; c World Health Organization, World Health Organization Headquarters, Avenue Appia, 20, 1211, Geneve, Switzerland; d Ministry of Health, P.O. Box: 30016–00100, Nairobi, Kenya; e City Cancer Challenge (C/Can), Rue du Commerce 9, 1204, Genève, Switzerland; f Department of Surgery, School of Medicine, University of Washington, 1959 NE Pacific Street, Seattle, WA, 98195, USA; g Department of Global Health, School of Public Health, University of Washington, Hans Rosling Center, 3980 15th Ave NE, Seattle, 98190, WA, USA; h Health, Nutrition, Population Practice, World Bank, 1818 H St. NW, Washington, 20001, DC, USA

**Keywords:** Breast cancer, Economic evaluation, UHC

## Abstract

**Background:**

Cancer is the third leading cause of death in Kenya. Breast cancer is responsible for 3100 deaths annually. Quantifying the economic and social impacts of breast cancer supports inclusion of cancer care within Kenya's universal healthcare plan.

**Methods:**

Kenya's Ministry of Health led an economic cost-benefit analysis of expanding breast cancer prevention and treatment services. Three scenarios (early diagnosis only, screening with clinical breast exam (CBE-led), and screening with mammography (MG-led)) were modelled using an adapted version of a deterministic state-transition cohort simulation model jointly developed by the World Health Organization (WHO) and the International Agency for Research on Cancer (IARC) and maintained by Forecast Health. Real world evidence on the favorable stage-shift induced by each early detection scenario was used as model inputs. The model estimated the mortality benefits of favorable stage-shifting, and net financial costs and health and economic benefits in 2020 USD.

**Findings:**

Respectively, over 40 years, the cost to sustain early diagnosis programs only, CBE-led screening, or mammogram-led screening would require 1.4, 2.8, or 5.2 percent increases above current government health spending. All three strategies are economically efficient in the long run. Net economic benefits of expanded breast cancer care using clinical breast exam screening are $2.3 billion dollars (USD) over the next 40 years with 236,000 women's lives saved in Kenya. Mammographic screening provides net benefits of $1.9 billion (USD) with an additional 34,000 lives saved over 40 years compared to the CBE-led screening approach. Over 40 years, an early diagnosis-only strategy saves the fewest lives and has the lowest net benefit among the three strategies.

**Interpretation:**

We offer a novel economic evaluation for breast cancer prevention and care expansion within Universal Health Coverage in Kenya. It demonstrates the economic viability of providing those services in a low-middle income (LMI) context.

**Funding:**

The work was funded by the World Bank Group’s Tackling Non-Communicable Diseases Challenges in Low- and Middle-Income Countries Trust Fund, supported by the Access Accelerated Partnership. This report was also partially financed by the Global Financing Facility for Women, Children and Adolescents (GFF). The GFF is a global multi-stakeholder partnership hosted at the World Bank that provides catalytic financing and technical support for safe and equitable delivery of essential health and nutrition services for women, children and adolescents, while helping countries to build more resilient health systems.

**Research in context**

**Evidence before this study**

In recent years, authors have pointed out “a lack of a coherent economic case” for investing in cancer control in low-middle-income countries (LMICs), issued calls for “an evidence-informed” breast cancer investment case and pointed to a significant “survival gap” between LMICs and high-income countries. A global analysis of economic costs and benefits for 11 cancers was published by *The Lancet* in 2021. The World Health Organization (WHO) initiated development of a population-based cancer economic model in 2018 that has been used in Mozambique but no published studies from individual LMICs are yet available.

**Added value of this study**

This economic evaluation describes a pathway to improve breast cancer care under significant resource constraints. It is the first application in a country of a WHO-sponsored model that is available for use by other countries.

**Implications of all the available evidence**

Countries with limited resources can reduce breast cancer morbidity and mortality with early diagnosis and population screening techniques scaled to available treatment capacity.

## Introduction

Breast cancer is responsible for 11 percent of total cancer-attributable mortality in Kenya and is the second-largest cause of cancer mortality after cancer of the cervix.^1^ In 2023, Kenya launched a Cancer Control Strategic Plan to expand access to cancer services within Kenya's implementation of universal health coverage (UHC).^2^ Cancer prevention, early cancer detection, and effective multimodality treatment are central elements of the Plan. The National Cancer Control Program (NCCP) prepared a first-ever national cancer investment case to a) inform priority-setting for the cancer benefit package, and b) identify resources required to sustainably scale-up cancer services following the strategic plan.^3^

Low breast cancer survival rates are largely caused by patient and health system barriers that obstruct early cancer detection efforts, diagnosis, and treatment. Early cancer detection aims to stage-shift through public breast cancer education, early diagnosis, and screening. Early diagnosis aims to get women with breast abnormalities to receive accurate and prompt breast cancer diagnosis in primary health clinics. Screening entails testing women without clinical symptoms of breast pathology. Screening can be done with mammograms or clinical breast exam, which have differing implications for stage-shifting, mortality and cost.

Service uptake is limited among patients by low awareness, preferences for traditional forms of healing, distrust of the health system, gender dynamics, household responsibilities, and stigma.^4^, ^5^, ^6^, ^7^ In a coastal community in southeast Kenya, for example, only one in four surveyed women knew any breast cancer signs or symptoms.^8^ In western Kenya, women's knowledge of early-stage symptoms was found to be less than that of late-stage symptoms.^9^ On the health system side, barriers include low provider awareness of symptoms, long waiting times at public health facilities, access challenges for rural residents, insufficiency of trained technicians and specialists, and lack of adequate facilities and equipment.^5^^,^^6^

Kenya's stated goal is to increase breast cancer screening from 14 percent in 2021 to 30 percent by 2027.^2^ The higher patient volume implied will require substantially more health system capacity. In contexts where health systems may be challenged by such loads, the WHO recommends an “early diagnosis (ED) first” strategy in which women are educated about early breast cancer symptoms (small masses, subtle thickenings) and encouraged to present promptly to the health system for diagnostic evaluation.^10^ Compared to population-wide screening programs, ED programming can reduce the number of women requiring diagnostic workups by 10-fold,^10^ allowing time for mandatory health system diagnostic, pathology, and treatment services to establish or strengthen without being overwhelmed.

Mammographic (MG) screening remains the gold standard to facilitate early cancer detection, forming the backbone of population-level screening programs in well-resourced settings that have contributed to rates of early or localized stage detection as high as 70 percent.^11^ However, resource limitations may preclude widespread use of mammogram for screening in LMICs.^12^ For example, despite recently increasing the number of mammogram machines in Kenyan government hospitals from 11 to 50, use remains low. Reasons include low breast cancer awareness, significant out-of-pocket costs for mammography services, and few trained radiologists and radiology technicians.^13^ Even in systems where mammographic screening is well established, nearly half of breast cancers are diagnosed clinically rather than through screening programs.^10^

Screening administered through clinical breast exams (CBE) is an option in contexts where capacity for mammographic screening is building. Global evidence has shown that in previously unscreened populations where advanced stage cancers are common, clinical breast exams are highly effective in finding the same cancers as detected on the initial rounds of mammogram screening, with well-trained health providers performing CBE able to detect nine of 10 prevalent breast cancer cases.^14^

## Methods

This analysis considers the costs and benefits of an “ED first” approach, followed by the scale up of either CBE-led or MG-led breast cancer screening. The analysis uses a 40-year time horizon to capture lifetime impacts. The scale-up of screening and care is assumed to occur in three sequential phases.

- A five-year period (2022–2027) with ED-only to allow for health system capacity to grow;
- A ten-year period (2028–2037) of screening with one of the two screening techniques (CBE or MG) and continued health system strengthening;
- A 24-year period (2038–2061) of capacity development that results in a mature, sustainable screening and diagnosis program with both CBE and MG.

All costs and monetized benefits are enumerated in 2020 United States dollars after converting from Kenyan Shilling using a 106:1 exchange rate.^15^ Future costs and benefits are discounted at a rate of five percent.^16^ Table 1 describes model inputs, data, and sources.

**Table 1:** Summary of key model inputs, point estimates, and sources.

Parameter	Point estimate	Source(s)/notes
**Costs per unit—Early cancer detection and treatment**		
Clinical breast exam	USD 1.9	World Health Organization (WHO) CHOICE—reflects the cost of the average visit to a level 1–3 health facility in Kenya.^17^
Core biopsy + histology	USD 37.8	WHO Cancer Team, WHO CHOICE, and Atieno et al. (2018)—reflects equipment, supply, health facility visit costs, and laboratory costs. Equipment and supply costs were shared by the WHO Cancer Team and the cost of a 100-min visit (length derived from the UN Interagency One Health Tool) at a hospital facility was applied from WHO CHOICE.^17^ Histology costs are from Table 4 of Atieno et al. (2018), updated to 2020 KES.
Mammogram	USD 19.5	Mwenda et al. (2021)—authors' report of KES 2000 updated to 2020 KES and converted to USD at the period exchange rate (106:1)^18^
Health system strengthening (i.e. program) costs per person screened	USD 16.1	Kenya Breast Cancer Action Plan—Derived from MoH estimates to fulfill its Breast Cancer Action Plan, covering five key areas. These included 1) governance and policy; 2) demand creation through community education and engagement; 3) health provider training and professional development; 4) service delivery (screening, diagnostics, patient navigation, and referral), and 5) monitoring and evaluation. We mapped costs to the number of women MoH expected to reach as a result of the program to create a unit cost per person screened.^19^
Palliative care	USD 1132.7	Atieno et al. (2018)—authors' estimate (Table 2) updated to 2020 KES and converted to USD at the period exchange rate.^20^^,^^21^
Treatment: Stage 1 breast cancer	USD 424.9	Atieno et al. (2018)—authors' estimates (Table 2, Table 4, Table 6) updated to 2020 KES and converted to USD at the period exchange rate. The cost reflects a weighted average of the estimated USD 309.1 cost for a radical mastectomy and USD 1439.0 for chemotherapy following the OneHealth Tool's assumptions that 100% of patients receive a radical mastectomy and 5% receive chemotherapy.^20^ Also, following OneHealth Tool assumptions 100% receive laboratory tests—complete blood count and liver and renal function tests (respectively USD 5.7,10.3,14.9) and 40 percent receive hormone therapy (tamoxifen, annually USD 56.2).
Treatment: Stage 2 breast cancer	USD 789.2	Atieno et al. (2018), authors' public sector updated to 2020 KES, reflecting a weighted average of radical mastectomy and chemotherapy costs assuming that 100% of patients receive a radical mastectomy, 30% receive chemotherapy, 100% receive laboratory tests, and 50 percent receive hormone therapy.
Treatment: Stage 3 breast cancer	USD 1220.9	Atieno et al. (2018), authors' public sector estimates updated to 2020 KES, reflecting a weighted average of radical mastectomy and chemotherapy costs assuming that 100% of patients receive a radical mastectomy, 60% receive chemotherapy, 100% receive laboratory tests, and 50 percent receive hormone therapy.
Treatment: Stage 4 breast cancer	USD 1249.9	Atieno et al. (2018), authors' public sector estimates updated to 2020 KES, reflecting a weighted average of radical mastectomy and chemotherapy costs assuming that 20% of patients receive a radical mastectomy, 80% receive chemotherapy, 100% receive laboratory tests, and 25 percent receive hormone therapy.
Ultrasound	USD 19.3	Kenyatta National Hospital charge rate, updated to 2020 KES and converted to USD at the period exchange rate.^22^
**Demographic and epidemiological**		
Breast cancer diagnosis, # of women	6451	Globocan—Cancer Today 2020 (see also Appendix Table A1 for inputs by age group)
Breast cancer prevalence (5-year), # of women	15,496	Globocan—Cancer Today 2020 (see also Appendix Table A1 for inputs by age group)
Healthy population of women aged 0 to 100	25.2 million	United Nations Population Division estimates. Demographic projections within the model based on DemProj, the foundation for Spectrum's health modules^23^ (see also Appendix Table A1 for inputs by age group).
Healthy population background annual mortality rate of women (%)	0.37%	United Nations Population Division (see also Appendix Table A1 for inputs by age group)
Stage at diagnosis, proportion of all women diagnosed with breast cancer	Stage 1–0.05 Stage 2–0.26 Stage 3–0.31 Stage 4–0.38	Kenya Breast Cancer Screening and Early Diagnosis Action Plan
**Economic**		
Discount rate	0.05	Haaker et al. (2020).^24^
Exchange rate: 2020 Kenyan Shillings to United States Dollars	106.5: 1	Period average exchange rate—The World Bank Database^25^
Government Health Expenditures/government NCD expenditures	USD 2.0 billion/494.4 million	WHO Health Expenditures database.^26^
Projected real GDP growth rate (%)	3.9	Based on historic and projected data from the International Monetary Fund's World Economic Outlook dataset^27^
Value of a statistical life (VSL) year	USD 1431.5	Derived from a United States VSL estimate (USD 11.6 million)^28^ following methodological guidance from Reference Case Guidelines for Benefit-cost Analysis and using the advised income elasticity of 1.5.^29^
**Scenario scale ups and model effect sizes**		
Early awareness campaign downstaging effect—proportion of women diagnosed with early stage 1 or 2 cancer	0.31–0.50	Based on observed effect in randomized controlled trials in Kenya and India: Mittra et al. (2010) and Pace et al. (2019).^30^^,^^31^
Clinical-breast examination-led screening downstaging effect—proportion of women diagnosed with early stage 1 or 2 cancer	0.50–0.63	Based on observed effect in a randomized controlled trial in India: Mittra et al. (2010).^31^
Mammogram-led screening downstaging effect	0.50–0.69	Verdial et al. (2017)–Based on historic data from the United States evidencing early cancer detection rates pre- and-post 10-year scale up of mammographic screening.^32^
Scale up (baseline/target) of screening from 2038-2037—the percent of all women age 40 to 74 who are screening for breast cancer in relevant scenarios	1/70	Baseline rate obtained from Kenya's Cancer Policy 2019–3030.^33^ Target is reflective of screening programs in Europe,^34^ and is used as representative of the kind of coverage needed to achieve the downstaging effect of mature screening programs.
Scale up (baseline/target) of treatment from 2021 to 2030—the percent of all women diagnosed moving on to receive treatment	60/90	Baseline rates are estimates based on conversations with the Ministry of Health. Targets are mapped to goals specified in Kenya's Breast Cancer Screening and Early Diagnosis Action Plan to “ensure at least 90% of symptomatic women are linked to further evaluation and management”.(3, p.30)
Scale up of palliative care (baseline/target) from 2021 to 2030—the percent of all cancer patients in need of palliative care who access it	42/80	Baseline and target rates are from Kenya's Cancer Policy 2019–2030.^33^
Sensitivity/specificity of breast cancer diagnosis tests (%)	Clinical breast exams (CBE)—60/95 Mammogram—87/89 Ultrasound—80/88 Core needle biopsy—87/98	CBE^35^; Mammogram^36^; Ultrasound^37^; Core needle biopsy.^38^ To calculate the number of women who were administered screening and diagnostic services, the model started with the number of women who were diagnosed with breast cancer each year and back-calculated the expected number of women who would have had to proceed through screening and diagnosis pathways—using known accuracy of diagnostic tests.
Survival rates with/without treatment, by stage	Stage 1–0.97/0.85 Stage 2–0.95/0.68 Stage 3–0.76/0.28 Stage 4–0.21/0.18	Survival rates with and without cancer are informed by Gopalappa et al. (2018).^39^ Within the model, persons who survived after five years were considered “recovered” and were subject to background mortality rates equivalent to those in the healthy population.
**Other**		
Number of screens per year on one mammogram machine	5800	Bansal et al. (2020)^40^—used to assess the number of mammogram machines required in scenarios where screening scales (see Appendix 2 section 2.3)

### Model and data

The analysis used an adapted version of a deterministic state-transition cohort simulation model jointly developed by WHO and IARC using version 1.0 of the Forecast Health Modelling Platform (Figure A1). The original model is described in earlier sources, including handling of demographic projections, estimation of health outcomes, health state transitions based on calculated population risks.^41^, ^42^, ^43^

The model uses Kenya-specific incidence and mortality data from Globocan Cancer Today and Globocan Cancer Tomorrow^1^ and distributes the population of women aged 40–74 years into initial health states. Rates of existing screening, diagnosis, and treatment, as well as scale up goals, were obtained from national data sources in collaboration with the Kenya Ministry of Health. The stage-shifting effect of early diagnosis, and population-wide CBE- or MG-led screening programs, as well as mortality reduction effects of treatment, are drawn from published and Kenya-relevant literature.^44^, ^45^, ^46^, ^47^, ^48^, ^49^

### Description of scenarios

In scenario 1 (“ED-only”), a “pre-screening” phase, Kenya implements a phased early diagnosis program for symptomatic women (2022–2027). National-level education and outreach campaigns support women to recognize early-stage breast cancer symptoms and present at primary care facilities for CBE. Complementary investments are made to train primary healthcare workers in best-practice clinical breast examination. In scenario 1, Kenya's ED-only program is continued with no national-level screening program.

In scenarios 2a and 2b, after five years of ED-only, population level screening is initiated and scaled over a ten-year period (2028–2037) in asymptomatic women age 40–74. It includes personalized screening-by-appointment, breast cancer awareness campaigns and mobile clinic outreach in targeted community or workplace settings. Two different screening technologies are modeled. Scenario 2a is “CBE-led” with screening conducted through clinical breast exam. Scenario 2b is “MG-led” with mammography screening for women ages 40–74. In both scenarios, 70 percent coverage is achieved by 2037, reflective of rates achieved by mature screening programs in Europe.^50^

**Fig. 1:**
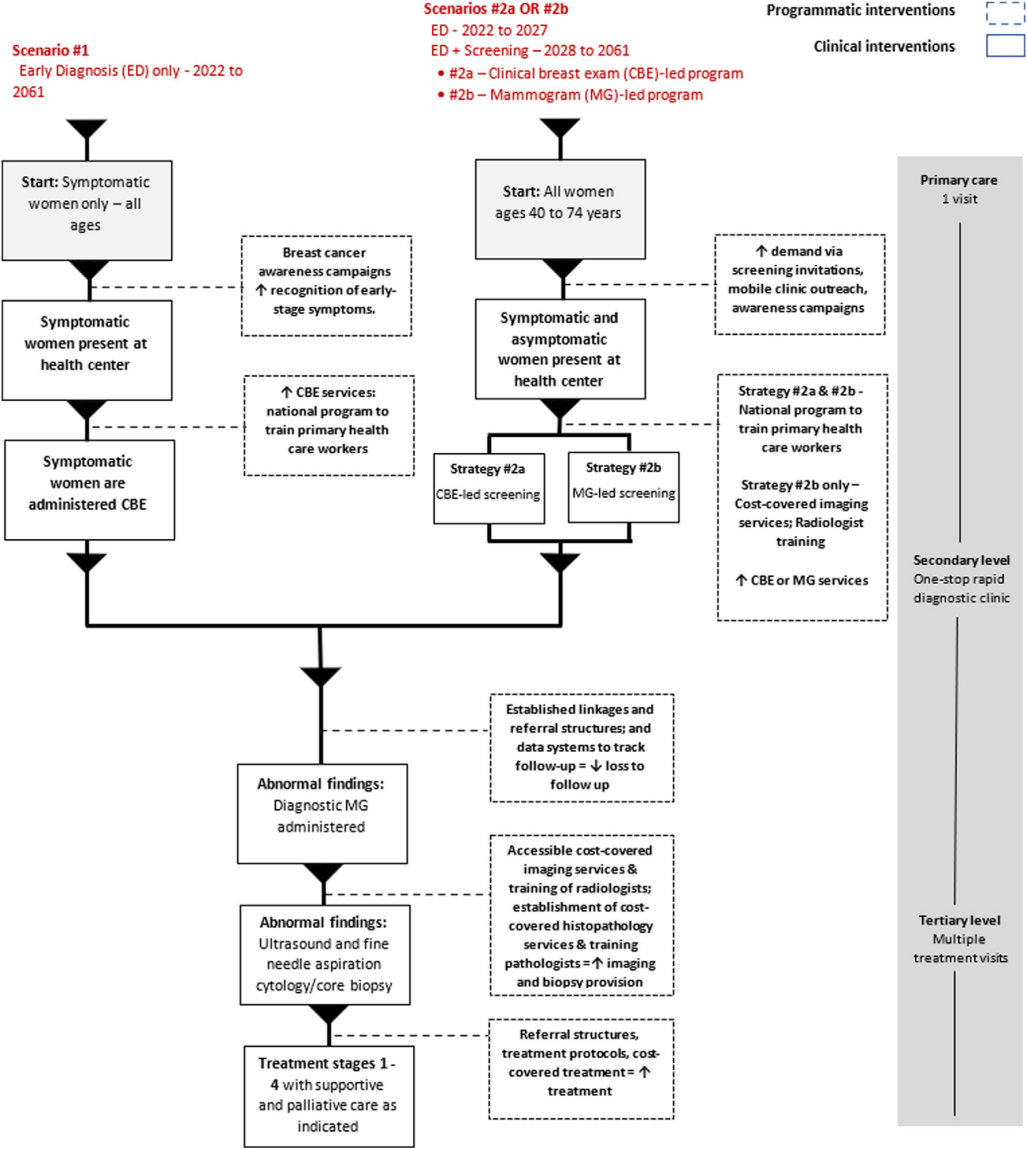
**Scenario pathways**.

In all scenarios, patients with abnormal findings discovered at the primary care level will be referred to secondary levels of care. Clinically indicated treatment for stage one to four cancer is available for diagnosed cases. Following the goals stated in Kenya's Breast Cancer Action Plan,^13^ the intervention scenarios assume that cancer treatment initiation rates increase linearly from 60 to 90 percent and that palliative care coverage grows from 42 to 80 percent from 2021 to 2030. Fig. 1 visualizes the pathway of each scenario and health systems strengthening efforts that support the scale up of interventions supporting breast cancer control.

### Modeled intervention effects

The “status quo” scenario describes the expected health burden if Kenya does not take any additional action to improve early cancer detection and treatment of breast cancer. In this scenario, throughout the time horizon of the analysis, early cancer detection rates remain stagnant at 31 percent, only 60 percent of all diagnosed women move on to receive treatment, and only 42 percent of those in need of palliative care access it.

In the other scenarios, the interventions determine how rates of diagnosed cancer, the stages at diagnosis, and treatment rates differ from the status quo. The model applied outcomes from real-world observations of early clinical diagnosis and screening programs. In two randomized controlled trials (RCTs)^31^^,^^48^ ED-only led to between 47 percent (Rwanda) to 53 percent (Mumbai, India) of incident cases diagnosed at stage one or two. Based on those experiences, we assumed Kenya could increase early cancer detection rates from 31 to 47 percent in the first two years and achieve 50 percent after five years of a national-level ED program. In the next phase, CBE-led screening could grow early cancer detection rates to 63 percent based on results from the biennial CBE screening in the Mumbai trial, and MG-led screening could achieve 69 percent early-stage diagnosis based on historic data from the United States.^51^

All stage shifting occurs by detecting more women with early-stage cancer, while rates of late-stage detected breast cancer remain relatively unchanged. This assumption is consistent with global evidence that even high intensity screening programs will continue to have a baseline late-stage presentation that persists.^11^

### Health system costs

Costs are financial costs, or those actually paid to provide health services. While individuals, private payers, and others may incur a portion of the costs, the analysis frames costs as government investments that are required to strengthen the health system to provide care for breast cancer.

Government investment is required to: (i) scale prevention, screening, diagnosis, and treatment of breast cancer; and (ii) implement health systems strengthening measures to improve availability, affordability, and acceptability of care. Local per person screening, diagnosis, and treatment costs were primarily obtained from published studies and Ministry of Health (MoH) reports or plans.^52^^,^^53^ Where local cost data was unavailable, we drew on existing micro-costing frameworks developed by WHO to estimate the resources involved for a procedure and the per unit costs of those individual resources.^54^ Per person costs were applied to the number of women who receive an intervention—calculated using the prevalence of breast cancer, the sensitivity and specificity of administered tests, and the coverage rates of interventions—to reach total costs.

Health system strengthening costs were derived from existing Kenya MoH estimates for its Breast Cancer Action Plan (see Supplemental Appendix A, Table A2).^13^ Costs from the Plan covered five key areas: 1) governance and policy; 2) demand creation through community education and engagement; 3) health provider training and professional development; 4) service delivery (screening, diagnostics, patient navigation, and referral), and 5) monitoring and evaluation.

We estimated the percent increase in annual government health expenditures (GHE) required to fund each scenario. We drew annual GHE— USD $2 billion or USD $37 per person—from the WHO Health Expenditures database^55^ and projected government health expenditures in future years assuming static per person expenditure (total expenditures increase due to a growing population). We then compared total undiscounted GHE in each scenario.

### Monetizing improvements in health

Improvements in health (i.e. reduced mortality) were monetized using a “value of a statistical life year” (VSLY) measure. Following guidance from the Reference Case Guidelines for Benefit-cost Analysis,^56^ we estimated the value of one additional year of life for Kenya at USD $1241 and adjusted the value year-over-year for expected growth in real income. The projected VSLY in each year is multiplied by years of additional life to calculate the value of improvements in life expectancy. Benefit-cost ratios (BCR) are the discounted monetized value of averted mortality divided by the discounted sum of costs (i.e. health system strengthening and health-service delivery costs) to implement a given scenario.

### Role of the funding source

World Bank technical experts reviewed and commented on multiple drafts and are co-authors.

## Results

Table 2 reports incremental costs to scale breast cancer care compared to a status quo scenario in which no health system strengthening occurs and rates of service delivery do not change from the existing status quo. During the first five years, 61 percent of costs are used to prepare and strengthen the Kenyan health system to provide breast cancer services. Service delivery costs predominate over the medium-to long-term and are highest in scenarios in which Kenya scales national-level screening programs. Over 40 years, a mammogram-led screening program costs nearly twice as much as a CBE-led program, with the cost of screening forming nearly 39 percent of total costs compared to only six percent of costs in the clinical breast exam-led scenario. Over 40 years, Kenya would need to increase its annual government health expenditures by 1.4, 2.8, or 5.2 percent to fully fund the implementation and scale up of a program focused only on, respectively, early diagnosis, or clinical-breast-exam- or mammogram-based national-levels screening programs.

**Table 2:** Cumulative health system strengthening and breast cancer service delivery costs over 5, 15, and 40 years, by scenario (USD millions, discounted).

Cost category	5-year	15-year			40-year		
	All scenarios	ED-only	CBE-led screening	MG-led screening	ED-only	CBE-led screening	MG-led screening
Health systems strengthening^a^	36.8	97.2	175.9	189.3	276.9	589.8	643.0
Service delivery	23.8	107.0	157.0	357.1	268.1	474.4	1279.1
Screening	0.0	0.0	15.7	187.6	0.0	62.3	745.8
Diagnosis	4.1	14.5	33.2	50.8	33.2	114.8	190.6
Treatment	15.0	68.4	81.5	90.8	166.7	219.1	257.6
Palliative care	4.7	24.1	26.7	27.8	68.1	78.2	85.1
Total cost (required ↑ in annual GHE)^b^, ^c^	60.6 (0.5%)	204.2 (0.8%)	332.9 (1.5%)	546.4 (2.5%)	545.0 (1.4%)	1064.2 (2.8%)	1922.2 (5.2%)

a Health system strengthening costs consist of resources used to strengthen governance and policy, demand creation, train health providers, enhance service delivery, and implement monitoring and evaluation. A breakdown of costs by category over five years is in Appendix 2 section 2.1.

b Subtotals may not sum to total cost exactly due to rounding.

c Percent annual increase in government health expenditures (GHE) required to fully fund scale up of a given scenario (undiscounted).

**Fig. 2:**
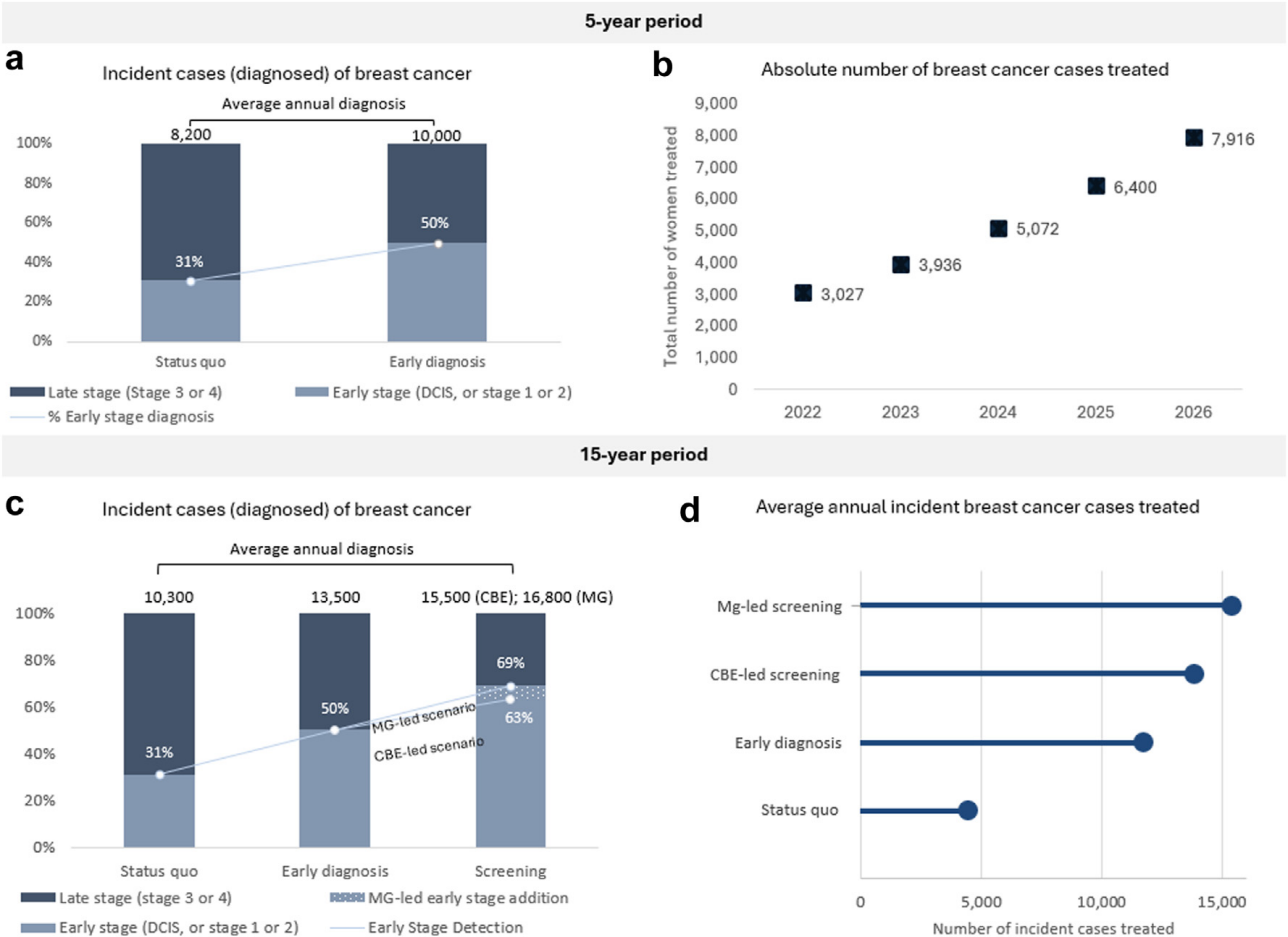
**Breast cancer cases diagnosed and treated over 5 and 15 years**.

As breast cancer control interventions scale, more women are diagnosed and more women receive treatment. Fig. 2a shows that over the first five years, a program focused on ED “uncovers” around 1800 more cases of breast cancer annually compared to if Kenya had not launched its breast cancer initiative. With treatment rates scaling simultaneously, the absolute number of women with incident cases who receive breast cancer treatment more than doubles from around 3000 in 2022 to 7900 in 2026 (Fig. 2b). Fig. 2c and d shows how caseload continues to increase as screening programs come online. Over a 15-year period, respectively, the CBE- and MG-led screening scenarios identify 2000 and 3300 more cases annually than if Kenya continued with an early diagnosis only (Fig. 2c). Compared to continuation of early diagnosis, the CBE-led screening scenario increases treated incident cases by 18 percent and the MG-led scenario increases treated incident cases by 31 percent (Fig. 2d).

**Fig. 3:**
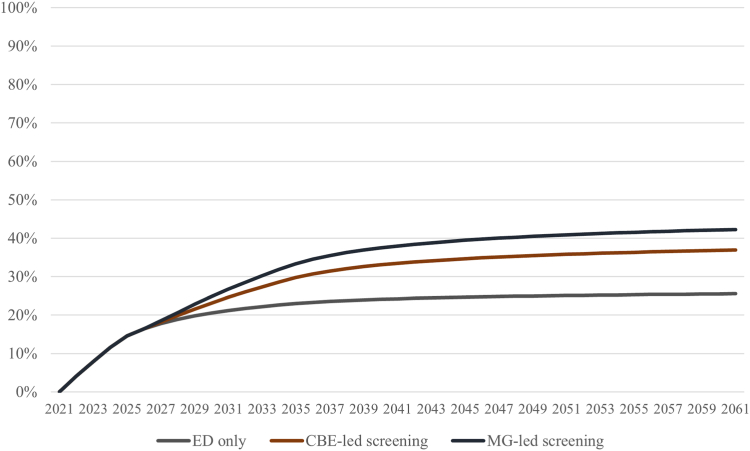
**Percent reduction in breast cancer-attributable deaths compared to continuation of status quo levels of service delivery**.

All scenarios significantly bend the projected mortality curve over the 40-year time horizon. Fig. 3 shows that over five years, with ED in place, breast cancer-attributable deaths decrease by around 15 percent (4700 lives saved) compared to the status quo scenario where Kenya maintains existing rates of service delivery. Over 40 years, the ED-scenario reduces deaths by 25.6 percent (163,000 lives saved), and the screening scenarios reduce deaths by 36.9 percent with CBE-led (236,000 lives saved) and 42.3 percent with MG-led (270,000 lives saved).

Table 3 summarizes the health-economic outcomes for the three scenarios. In the ED-only scenario, benefits of the strategy begin to outweigh costs (break-even point) in 2035, and the benefit-cost ratio (BCR) reaches 4.4 by the end of the model period (net benefits USD $1833 million). CBE-led screening breaks even in 2040, with a BCR of 3.2 by analysis end (USD $2298 million). MG-led screening breaks even in 2047, with a 2.0 BCR by analysis end (USD $1910 million). Over the 40-year time horizon, costs per DALY averted range between USD $729 for the ED only scenario and 1612 for the MG-led screening scenario.

**Table 3:** Summary of primary outcomes across the three modelled breast cancer scenarios—improvements in health, costs, monetized benefits, and summary measures (USD millions).

	Lives saved	Cumulative costs	Cumulative monetized benefits	Net benefits	Benefit-cost ratio	Cost per DALY averted
**Five years**						
All scenarios	4700	61	16	−45	0.26	18,194
**15 years**						
Early diagnosis only	33,600	204	233	29	1.2	2697
CBE-led screening	44,900	333	280	−53	0.84	3874
MG-led screening	50,600	546	307	−240	0.56	5937
**40 years**						
Early diagnosis only	163,200	545	2378	1833	4.4	729
CBE-led screening	235,800	1064	3362	2298	3.2	1015
MG-led screening	269,800	1922	3832	1910	2.0	1612

## Discussion

As Kenya seeks to fulfill its universal healthcare agenda, it will be balancing available resources against delivering optimal health services for multiple diseases and conditions. This analysis provides evidence to help meet that challenge, lending insight on pathways to strengthening care for breast cancer.

Examining the costs, benefits, and tradeoffs of differing pathways, we found that investment in activities to support ED (e.g., health worker training, national-level education and outreach campaigns), alongside Kenya's focus on scaling up treatment, could reduce expected breast cancer-attributable deaths by about 15 percent over five years, saving 4700 lives. In our analysis, achieving these health gains requires a health system prepared to respond to around 20 percent more cases of breast cancer identified and approximately double the incident cases needing treatment in five years.

The challenge of advancing early diagnosis at a pace matching health system preparedness has precedent in Kenya's context. When a breast cancer awareness campaign pilot in Nyeri County generated more than a six-fold increase in mammography services needed,^13^ existing health workforce and machinery were challenged to keep up. Back-ups and delayed reporting of results contributed to 40 percent of eligible patients not presenting for additional needed diagnostic work.^57^ Emphasizing ED first allows time to prepare health systems for increased service demand. Simultaneously, Kenya can develop primary-care provider expertise, increase imaging capacity, develop strong referral pathways, and ensure timely treatment is in place—and patient financial obstacles are removed—so that women who are diagnosed can access a health system ready to meet their needs. Few established breast cancer programs worldwide have achieved successful screening programs without first changing population knowledge and perceptions around breast cancer.^58^ Early diagnosis programs also lay the groundwork for screening.

After implementing a successful early diagnosis program, Kenya may choose to continue it without formal screening, or it may scale a population-level screening program. A mammography-led screening strategy would require funding equivalent to a 5.1 percent increase in annual government health expenditures while a CBE-led strategy would require around a 2.8 percent increase. When compared to European countries, where the care for *all* cancers represents between three and seven percent of health expenditures,^59^ resources are a challenge. Mammographic screening requires large investments in machines and maintenance-capacity as well as radiologists, imaging technologists and other personnel. To meet the needs of its population, Kenya would need to increase availability of mammogram machines from less than one machine per million people to a minimum of 21 machines per million people (Appendix 2 section 2.3)—with a proportionate expansion of human resource capacity.

A mammogram-led approach provides incremental improvements (an additional 34,000 lives saved over 40 years compared to the CBE approach) that must be weighed against needs for other important health services under Kenya's universal healthcare platform that may have greater potential to save lives on a per shilling basis. Each strategy is economically efficient in the long run. Over 40 years, the ED-only strategy has the highest BCR (4.4) but saves the fewest lives and has the lowest net benefit among the three strategies. The CBE-led screening strategy has a BCR of 3.2 and generates net benefits of USD $2298 million. The MG-led screening scenario has a BCR of 2.0 and net benefits of USD $1910 million.

All projected outcomes are dependent on at least 90% of patients receiving treatment. In places where appropriate referral pathways are not in place to assist women to navigate the health system, or where diagnosis and treatment is not accessible, affordable, and/or acceptable, public confidence is undermined.^60^ If the treatment threshold is not achieved, then Kenya may see favorable stage shifting but might not see the projected mortality benefit.

Our analysis benefits from several strengths and is also checked by limitations. A strength is incorporating data from the significant body of cancer research that already exists in Kenya. The analysis used in-country costing studies by the MoH and academic researchers, drew on national scale-up plans and treatment algorithms to create scenarios, and used data on the existing state of the health system to contextualize the scenario pathways. Further, the assumed impact of each strategy is drawn from real-world outcomes from studies conducted in Rwanda and India. Finally, we closely followed reference guides outlining accepted methods for economic evaluations of health programs.

Regarding limitations, the analysis uses modelled epidemiological data based on two Kenyan cancer registries covering about 11 percent of the national population, which is below the recommended 20 percent coverage for a high-quality population-based cancer registry. The limited geographic scope of the cancer registries means that the modelled epidemiological data may not represent the true burden of breast and cervical cancer in Kenya. Still, the modelled data is the best available describing the breast cancer burden at the national level.

We acknowledge uncertainty in estimates of costs and health and economic benefits. In some cases, they are undercounted. For example, while the modelling quantifies and monetizes mortality benefits (lives saved), morbidity was not quantified and monetized. Inclusion of morbidity benefits would be unlikely to materially change results. The costs of treatment may also be undercounted. The analysis used inputs for the costs of screening, diagnosing, and treating breast cancer from those estimated at public rather than private health facilities and hospitals—which are known to impose far higher costs than public facilities.^52^^,^^61^ We explored how using private sector costs would affect results. Substituting the few private-sector cost inputs we had available for stage I-IV treatment (ranging between 65 and 157 percent more than public sector costs) and palliative care (around 25 percent more than public sector costs), we found that the BCR for the CBE- and MG-led scenarios remained above one over 40 years, respectively decreasing to 2.5 and 1.7. We further explore the sensitivity of input parameters in the Supplemental Appendix, Fig A2.

Additionally, for costs, we assumed specific allocations of surgery and adjuvant therapy (chemotherapy, endocrine therapy); however, other treatment types such as radiotherapy and biological therapies such as trastuzumab were not included in the analysis due to uncertainty over changing health system responses and availability of advanced forms of treatment. Finally, the modelled costs and benefits are based on assumptions that Kenya will achieve its goals to scale early diagnosis programs, and particularly that nearly all Kenyan women who are diagnosed have access to—and receive—treatment. Results should be interpreted accordingly as reflective of what is *possible* should the Ministry be successful in implementing each step of a breast cancer control program with appropriate levels of quality.

This study did not explore the implications for people who do not have regular contact with the health system and are otherwise excluded, socially or economically, from accessing services. Equity must be considered when applying these results to decision-making. This study also did not address specific groups within the population who may have higher risk of breast cancer (e.g., those with a family history or genetic mutations). Higher levels of resource use may be needed to screen these populations, particularly those outside of the screening age range assessed in this analysis.

Our analysis of breast cancer care expansion pathways in Kenya highlights tradeoffs in resource use, costs, and health outcomes when considering more versus less intensive early cancer detection programs. Investments in breast cancer control save lives and provide a return on investment. But a realistic build-up of necessary resources to close health system capacity gaps is required to implement the breast cancer plan. Many countries are currently establishing cancer control programs and goals and will benefit from the evidence developed for Kenya.

The investment case was prepared to support deliberative decision-making among government agencies. The Ministry of Health intends to use the analysis results to plan for Kenya's next Medium-Term Expenditure Framework in 2026—giving it an ability to present clear budgetary impacts of its programmatic strategies.

## Contributors

MN, TLK, and RN conceived the study. BH, RW, BOA, AI, FP developed the model, conducted the analysis, and interpreted the results. JC, EW, VM, RJ, PY, KMK, AGT, FM, and CM provided review on specific model inputs and structure. BH wrote the first draft. RN wrote the second draft with substantial input from BH, BOA, MN, TLK, PY, AGT, and KMK. All authors reviewed and provided input to the submitted manuscript.

## Data sharing statement

Available upon request from WHO at ailbawi@who.int.

## Declaration of interests

RN has no conflicts of interest. She reports consulting fees from the World Bank in association with preparation of this manuscript and NCD economic analyses. Filip Meheus reports travel fees from Penn Dental Medicine. All authors declare that they have no conflicts.

## References

[1] GLOBOCAN 2020 Database (2022). http://gco.iarc.fr/today/home.

[2] Kenya Ministry of Health (2022). Kenya national cancer control strategy, 2023-2027.

[3] World Bank (2022).

[4] Muthoni A., Miller A.N. (2010). An exploration of rural and urban Kenyan women's knowledge and attitudes regarding breast cancer and breast cancer early detection measures. Health Care Women Int.

[5] Gakunga R., Kinyanjui A., Ali Z. (2019). Identifying barriers and facilitators to breast cancer early detection and subsequent treatment engagement in Kenya: a qualitative approach. Oncol.

[6] Antabe R., Kansanga M., Sano Y., Kyeremeh E., Galaa Y. (2020). Utilization of breast cancer screening in Kenya: what are the determinants?. BMC Health Serv Res.

[7] Zolnikov T.R., Chambers D. (2019). Barriers to treatment for patients with breast cancer in Kenya. Lancet Oncol.

[8] Sayed S., Ngugi A.K., Mahoney M.R. (2019). Breast cancer knowledge, perceptions and practices in a rural community in coastal Kenya. BMC Publ Health.

[9] Naanyu V., Asirwa C.F., Wachira J. (2015). Lay perceptions of breast cancer in Western Kenya. World J Clin Oncol.

[10] (2017). Guide to cancer: early diagnosis.

[11] Verdial F.C., Etzioni R., Duggan C., Anderson B.O. (2017). Demographic changes in breast cancer incidence, stage at diagnosis and age associated with population-based mammographic screening. J Surg Oncol.

[12] (2014). WHO position paper on mammography screening.

[13] Kenya Ministry of Health Breast cancer screening and early diagnosis action plan 2021-2025. Nairobi, Kenya. https://www.health.go.ke/wp-content/uploads/2021/10/Breast-Cancer-Screening-and-Early-Diagnosis-Action-Plan-2021-2025.pdf.

[14] Ngan T.T., Nguyen N.T.Q., VanMinh H., Donnelly M., O'Neill C. (2020). Effectiveness of clinical breast examination as a “stand-alone” screening modality: an overview of systematic reviews. BMC Cancer.

[15] World Development Indicators DataBank. https://databank.worldbank.org/source/world-development-indicators.

[16] Haacker M., Hallett T.B., Atun R. (2020). On discount rates for economic evaluations in global health. Health Pol Plann.

[17] Stenberg K., Lauer J.A., Gkountouras G., Fitzpatrick C., Stanciole A. (2018). Econometric estimation of WHO-CHOICE country-specific costs for inpatient and outpatient health service delivery. Cost Eff Resour Allocation.

[18] Mwenda V., Bor J.P., Gitungo H. (2021). Breast health awareness campaign and screening pilot in a Kenyan County: findings and lessons. Cancer Rep.

[19] Kenya Ministry of Health (2021). http://guidelines.health.go.ke/#/category/7/401/meta.

[20] Atieno O.M., Opanga S., Martin A., Kurdi A., Godman B. (2018). Pilot study assessing the direct medical cost of treating patients with cancer in Kenya; findings and implications for the future. J Med Econ.

[21] World Health Organization OneHealth Tool. Cost effectiveness and strategic planning (WHO-CHOICE). http://www.who.int/choice/onehealthtool.

[22] Kenyatta National Hospital (2016). https://knh.or.ke/wp-content/uploads/2017/03/KNH-Service-Charter-FINAL-DraftB.pdf.

[23] Avenir Health Spectrum models. https://avenirhealth.org/software-spectrummodels.php.

[24] Haacker M., Hallett T.B., Atun R. (2020). On discount rates for economic evaluations in global health. Health Pol Plann.

[25] World Bank (2021). https://databank.worldbank.org/source/world-development-indicators.

[26] World Health Organization (2020). https://apps.who.int/nha/database.

[27] International Monetary Fund Real GDP growth - annual percent change. World Economic Outlook Dataset. https://www.imf.org/external/datamapper/NGDP_RPCH@WEO/OEMDC/ADVEC/WEOWORLD.

[28] U.S. Department of Transportation (2021). https://www.transportation.gov/office-policy/transportation-policy/revised-departmental-guidance-on-valuation-of-a-statistical-life-in-economic-analysis.

[29] Robinson L.A., Hammitt J.K., Cecchini M. (2019). Reference case Guidelines for benefit-cost analysis in global health and development. SSRN Electron J.

[30] Pace L.E., Dusengimana J.M.V., Shulman L.N. (2019). Cluster randomized trial to facilitate breast cancer early diagnosis in a rural district of Rwanda. J Glob Oncol.

[31] Mittra I., Mishra G.A., Singh S. (2010). A cluster randomized, controlled trial of breast and cervix cancer screening in Mumbai, India: methodology and interim results after three rounds of screening. Int J Cancer.

[32] Verdial F.C., Etzioni R., Duggan C., Anderson B.O. (2017). Demographic changes in breast cancer incidence, stage at diagnosis and age associated with population-based mammographic screening. J Surg Oncol.

[33] Kenya Ministry of Health (2019).

[34] Peintinger F. (2019). National breast screening programs across Europe. Breast Care Basel Switz.

[35] Ngan T.T., Nguyen N.T.Q., Van Minh H., Donnelly M., O'Neill C. (2020 Nov 9). Effectiveness of clinical breast examination as a “stand-alone” screening modality: an overview of systematic reviews. BMC Cancer.

[36] Ernster V.L., Barclay J., Kerlikowske K., Grady D., Henderson C. (1996). Incidence of and treatment for ductal carcinoma in situ of the breast. JAMA.

[37] Sood R., Rositch A.F., Shakoor D. (2019). Ultrasound for breast cancer detection globally: a systematic review and meta-analysis. J Glob Oncol.

[38] Wang M., He X., Chang Y., Sun G., Thabane L. (2017). A sensitivity and specificity comparison of fine needle aspiration cytology and core needle biopsy in evaluation of suspicious breast lesions: a systematic review and meta-analysis. Breast Edinb Scotl.

[39] Gopalappa C., Guo J., Meckoni P. (2018). A two-step markov processes approach for parameterization of cancer state-transition models for low- and middle-income countries. Med Decis Mak.

[40] Bansal S., Deshpande V., Zhao X. (2020). Analysis of mammography screening schedules under varying resource constraints for planning breast cancer control programs in low- and middle-income countries: a mathematical study. Med Decis Mak Int J Soc Med Decis Mak.

[41] Ralaidovy A.H., Gopalappa C., Ilbawi A., Pretorius C., Lauer J.A. (2018). Cost-effective interventions for breast cancer, cervical cancer, and colorectal cancer: new results from WHO-CHOICE. Cost Eff Resour Allocation.

[42] Lauer J.A., Röhrich K., Wirth H., Charette C., Gribble S., Murray C.J. (2003). Cost Eff Resour Alloc.

[43] Watts R. Botech Protocol for the simulation of state transition modles. n.d. https://forecasthealth.org/botech-protocol/.

[44] Groot M.T., Baltussen R., Uyl-de Groot C.A., Anderson B.O., Hortobágyi G.N. (2006). Costs and health effects of breast cancer interventions in epidemiologically different regions of Africa, North America, and Asia. Breast J.

[45] Perez E.A., Romond E.H., Suman V.J. (2014). Trastuzumab plus adjuvant chemotherapy for human epidermal growth factor receptor 2–positive breast cancer: planned joint analysis of overall survival from NSABP B-31 and NCCTG N9831. J Clin Oncol.

[46] Davies C., Pan H., Godwin J. (2013). Long-term effects of continuing adjuvant tamoxifen to 10 years versus stopping at 5 years after diagnosis of oestrogen receptor-positive breast cancer: ATLAS, a randomised trial. Lancet.

[47] Romanoff A., Constant T.H., Johnson K.M. (2017). Association of previous clinical breast examination with reduced delays and earlier-stage breast cancer diagnosis among women in Peru. JAMA Oncol.

[48] Pace L.E., Dusengimana J.M.V., Shulman L.N. (2019). Cluster randomized trial to facilitate breast cancer early diagnosis in a rural district of Rwanda. J Glob Oncol.

[49] Pace L.E., Dusengiman J.M.V., Keating N.L. (2018). Impact of breast cancer early detection training on Rwandan health workers' knowledge and skills. J Glob Oncol.

[50] Peintinger F. (2019). National breast screening programs across Europe. Breast Care.

[51] Kardinah D., Anderson B.O., Duggan C., Ali I.A., Thomas D.B. (2013). Short report: limited effectiveness of screening mammography in addition to clinical breast examination by trained nurse midwives in rural Jakarta, Indonesia. Int J Cancer.

[52] Atieno O.M., Opanga S., Martin A.P., Kurdi A., Godman B. (2018). Pilot study assessing the direct medical cost of treating patients with cancer in Kenya; findings and implications for the future. J Med Econ.

[53] (2019). Service delivery charter: kenyatta national hospital.

[54] UN InterAgency Working Group on Costing (IAWG-Costing) (2023). OneHealth tool. Avenir Health.

[55] (2020). Global health expenditure database.

[56] Robinson L.A., Hammitt J.K., Cecchini M. (2019). https://www.hsph.harvard.edu/wp-content/uploads/sites/2447/2019/05/BCA-Guidelines-May-2019.pdf.

[57] Kenya Ministry of Health, GE Healthcare, County Government of Nyeri (2020).

[58] Yip C.H., Smith R.A., Anderson B.O. (2008). Guideline implementation for breast healthcare in low- and middle-income countries: early detection resource allocation. Cancer.

[59] Hofmarcher T., Lindgren P., Wilking N., Jönsson B. (2020). The cost of cancer in Europe 2018. Eur J Cancer Oxf Engl.

[60] Ginsburg O., Yip C.H., Brooks A. (2020). Breast cancer early detection: a phased approach to implementation. Cancer.

[61] Subramanian S., Gakunga R., Kibachio J. (2018). Cost and affordability of non-communicable disease screening, diagnosis and treatment in Kenya: patient payments in the private and public sectors. PLoS One.

