# Supplementary material for: An economic evaluation of breast cancer interventions in Kenya
Source: eClinicalMedicine. 2024 Oct 30;77:102894. doi: 10.1016/j.eclinm.2024.102894 (PMC11563939; doi:10.1016/j.eclinm.2024.102894)
Supplement: Supplementary Appendix [file mmc1.docx]

# APPENDIX 1 – MODELLING AND DATA PARAMETERS

### **Overview**

Figure A1 describes the model: a dynamic state transition model designed to simulate the progression of breast cancer in Kenya over a 40-year period (2021-2061)—as modified by rates of detection and treatment. The model takes a graph structure - nodes and links - representing the key stages in breast cancer progression, diagnosis, treatment, and management, as well as intervention strategies such as screening.

**Figure A1. Breast cancer health-state transition model**


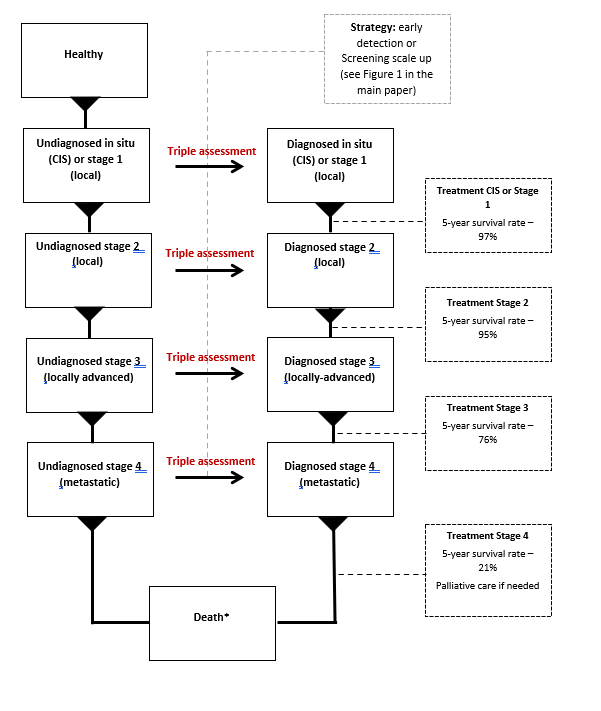


Abbreviations: CIS – Carcinoma in situ

5-year survival rates without treatment are 0.85, 0.68, 0.28, and 0.18 respectively for Stage 1-4 cancer

In the state transition model, each node holds a 2x101 array, with each element representing a sex and age from Males aged 0, to Females aged 100. The order of operations in the model is as follows: - Any necessary balances (e.g. the population of the country) are generated - Values are moved between states - Persons die - Persons age

The model employs a combination of file-based and synthetic data generation methods. The synthetic method is used extensively to simulate transitions between states, allowing for a dynamic representation of the disease progression and management in the Kenyan context. Data sources are detailed below, where relevant.

### States

Note - Generator is a node that is able to produce values. Note - Filter is a node that takes a proportion of another node’s values

1. **Population Generator:** Generates the population data for Kenya, utilizing the serve_population function from the UNDP World Population Projections.
2. **Incidence Filter:** Represents the incidence of breast cancer in the population, with incidence data by age and sex from IARC Globocan.
3. **Clinical Presentation Filter:** Accounts for breast cancer cases identified via clinical presentation. It filters cases based on diagnosis methods that exclude mammography (MG).
4. **Screening Filter:** Represents cases diagnosed through screening methods, including mammography.
5. **Diagnosed at Stage I-IV Filters:** Four separate filters categorizing patients diagnosed at different stages of breast cancer, each stage associated with a specific disability weight (0.29 for Stages I-III and 0.54 for Stage IV), taken from the Global Burden of Disease disability weights.
6. **Stage I-IV - Staging Filters:** Represent the staging process for each diagnosed stage, where patients are prepared for appropriate treatment.
7. **Stage I-IV - Treatment Filters:** Represent the treatment phase for each cancer stage.
8. **Stage I-IV - Management of Complications Filters:** Account for the management of treatment-related complications in each stage.
9. **Stage I-IV - Surveillance Filters:** Represent the surveillance phase post-treatment, monitoring for disease progression or recurrence.
10. **Mortality:** Represents the state of being deceased. An absorbing state. Transitions are either based on lifetable data from the Global Burden of Disease Study by age and sex, or by cancer specific mortality rates.
11. **Recovered:** Represents a person who has not died from cancer after receiving treatment for at least 5 years.

## Model data

**Table A1. Kenya Female Population – Annual status quo breast cancer incidence, prevalence, background mortality, by age**

| **Age group** | **Female population** | **Breast cancer prevalence** | **Annual incidence of breast cancer** | **Background mortality rate (annual)** |
| --- | --- | --- | --- | --- |
| 0 to 4 | 3,486,021 | 0 | 0 | 0∙02 |
| 5 to 9 | 3,451,279 | 0 | 0 | 0∙0008 |
| 10 to 14 | 3,340,913 | 5 | 0∙0000002 | 0∙0006 |
| 15 to 19 | 2,988,795 | 40 | 0∙000007 | 0∙001 |
| 20 to 24 | 2,617,391 | 7 | 0∙000002 | 0∙002 |
| 25 to 29 | 2,190,500 | 199 | 0∙00007 | 0∙003 |
| 30 to 34 | 203,004 | 611 | 0∙0002 | 0∙003 |
| 35 to 39 | 1,743,029 | 1,198 | 0∙0004 | 0∙004 |
| 40 to 44 | 1,397,049 | 1,730 | 0∙0006 | 0∙005 |
| 45 to 49 | 1,088,429 | 2,244 | 0∙0009 | 0∙006 |
| 50 to 54 | 821,975 | 2,519 | 0∙001 | 0∙008 |
| 55 to 59 | 627,839 | 2,180 | 0∙001 | 0∙01 |
| 60 to 64 | 477,352 | 1,883 | 0∙002 | 0∙02 |
| 65 to 69 | 351,130 | 1,465 | 0∙002 | 0∙02 |
| 70 to 74 | 220,032 | 891 | 0∙002 | 0∙04 |
| 75 to 79 | 116,868 | 383 | 0∙001 | 0∙07 |
| 80 to 84 | 65,195 | 129 | 0∙0008 | 0∙12 |
| 85+ | 28,932 | 12 | 0∙0002 | 0∙45 |

## Costing - Health systems strengthening costing

For the analysis, we estimated health systems strengthening costs required per woman screened (USD 16.1) using information in Kenya’s Breast Cancer Action Plan. Over a five-year initialization period, the plan costed governance and policy, demand creation and community education, training and professional development, service delivery, and monitoring and evaluation and research costs required to strengthen the health system to support a nation-wide screening program. Total costs of the 5-year plan are shown below in Fig A1. We divided total costs by the number of women expected to be screened under the plan (≈ 2.7 million) to roughly estimate health system strengthening investments required per additional women screened.

In the analysis’ MG-led screening scenario, we applied these per-person costs directly based on the additional of number of women screened. In the ED and CBE-led screening scenarios, we reduced health systems strengthening expenditures in proportion to the expected incidence under each scenario (i.e. if the early diagnosis program led to one-third of the identified breast cancer cases as in the MG-led scenario, we assumed health systems strengthening costs also dropped by one-third).

Table A1. Kenya Breast Cancer Action Plan – 5-year health system strengthening costs

## Sensitivity analysis

Table A2. Benefit cost ratio (BCR) of scenarios 2a and 2b in “Best” and “Worst” case scenarios

|  |  |  | **40-year BCR - Scenario 2a (Base case BCR = 3.16)** | | **40-year BCR - Scenario 2b (Base case BCR = 1.99)** | |  |
| --- | --- | --- | --- | --- | --- | --- | --- |
| **Input category 1** | **Input category 2** | **Input (point estimate)** | **Best case^a^** | **Worst case** | **Best case** | **Worst case** | |
| Cost | Screening and diagnosis | Clinical breast exam (USD 1∙9), Mammogram (USD 19∙5), Ultrasound (USD 19∙3), Core biopsy + histology (USD 37∙8) | 3∙34 | 2∙99 | 2∙38 | 1∙72 | |
| Cost | Treatment | Stage 1 (USD 424∙9), Stage 2 (USD 789∙2), Stage 3 (USD 1,220∙9), Stage 4 (USD 1,249∙9) | 3∙38 | 2∙97 | 2∙09 | 1∙91 | |
| Cost | Palliative care | USD 1,132∙7 | 3∙24 | 3∙08 | 2∙02 | 1∙96 | |
| Cost | Health systems strengthening | Cost per woman screened (USD 16∙1) | 3∙81 | 2∙7 | 2∙23 | 1∙81 | |
| Economic | Discount rate | 0∙05b | 3∙57 | 2∙78 | 2∙23 | 1∙77 | |
| Benefit | Value of a statistical life year | USD 1,4312∙5 | 4∙2 | 2∙65 | 2∙12 | 1∙34 | |
| All | -- | -- | 6∙98 | 1∙42 | 4∙41 | 0∙9 | |

1. For “Best” and “Worst” case scenarios, point estimates were varied up and down by 33 percent.
2. A lower discount rate (0∙0335) represented the best case scenario and a higher discount rate (0∙0665) a worst case scenarios

# APPENDIX 2 – Narrative-based results 🡫

**2.1. Phase 1 (2022 to 2027) –** Early diagnosis (ED) and health system strengthening (Pre-screening period)

**What’s happening?** – Kenya establishes foundational health system building blocks for a comprehensive cancer control program, focusing on health provider training (primary and secondary providers, and laboratory and imaging personnel) and service delivery (diagnostics, patient navigation, referral, and treatment). Focus is on early diagnosis, with sustained breast cancer awareness campaigns targeted to improve healthcare presentation rates among all women with breast cancer symptoms. Treatment rates scale from 40 to 64%. Palliative care from 42 to 59%. The government assesses the health system’s capacity to manage increases in demand and to ensure all diagnosed cases receive treatment before screening scale up places additional load on the system.

Average annual diagnosis

**Diagnosed breast cancer - percent by stage**

**5-year program & service delivery costs, USD million (discounted)**

**discounted**

| **Cost type** | **Total 5-year** | **Average annual** |
| --- | --- | --- |
| **Program** | **3,912** | **782** |
| Governance & policy | 50 | 10 |
| Demand creation | 357 | 71 |
| Health provider training | 771 | 154 |
| Service delivery | 2,584 | 517 |
| Monitoring & Evaluation | 149 | 30 |
| **Direct treatment costs** | **2,544** | **509** |
| Diagnosis | 418 | 84 |
| Treatment | 1,624 | 325 |
| Palliative | 502 | 100 |
| **Total** | **6,456** | **1,291** |

10,000

8,200

| **Cost type** | **Total 5-year** | **Average annual** |
| --- | --- | --- |
| **Health systems strengthening** | **36∙8** | **7∙4** |
| Governance & policy | 0∙5 | 0∙1 |
| Demand creation | 3∙4 | 0∙7 |
| Health provider training | 7∙2 | 1∙4 |
| Service delivery | 24∙3 | 4∙9 |
| Monitoring & Evaluation | 1∙4 | 0∙3 |
| **Direct treatment costs** | **23∙8** | **4∙8** |
| Diagnosis | 4∙1 | 0∙8 |
| Treatment | 15∙0 | 3∙0 |
| Palliative | 4∙7 | 0∙9 |
| **Total** | 60∙6 | 12∙1 |

Five-year program and service delivery costs are the same across all three scenarios

**Treatment for breast cancer**

**Breast cancer program launch**: *Demand creation -* awareness campaigns air on traditional and social media, with advocacy supported by community, religious, and other leaders; *Training* - health workers trained to increase breast cancer awareness and to administer CBE, training for imaging, lab, & health records personnel; *Service delivery* – establishment of eight breast cancer Centers of Excellence, imaging services in 47 counties, quality pathology services, and strong linkage and referral structures; and *M&E* - spaced, planned assessments.

**Costs:** Annual expenditures are a 0∙5 percent increase over existing levels of Kenyan government health expenditures (GHE)

**Diagnosis**: 8,100 more cases of breast cancer are diagnosed than if Kenya had not launched its breast cancer initiative.

**Treatment**: The number of women who receive breast cancer treatment more than doubles - 4,700 lives saved.

**5-year benefit-cost ratio (BCR)**: Early diagnosis (0∙3)

**Short-term outcomes of Early Diagnosis**

More women are diagnosed with breast cancer and treatment rates also scale among existing cases. By 2026, the health system requires capacity to treat more than 2x the number of breast cancer cases compared to if no intervention is put in place.

**2.2 Phase 2 (2028 to 2037) –** Screening scale up (CBE- or MG-led)

**What’s happening?** – Kenya continues with an ED-only approach in Scenario 1. In Scenarios 2a and 2b, Kenya determines that the health system is prepared to take on additional patient loads that will arise from population-level screening. It implements EITHER a biennial CBE- or MG-led invitation-based screening program for women age 40 to 74, based on a health system assessments. Over ten years, Kenya linearly scales an invitation-based screening program, reaching 70% of all women by 2037. Treatment and palliative-care rates continue to scale, so that 90 percent of all women diagnosed with breast cancer receive treatment and 80 percent of all individuals in need of supportive or palliative care receive it by 2030.

**Diagnosed breast cancer improvements - percent by stage**

Average annual diagnosis

**15-year program & service delivery costs, USD millions (discounted)**

| **Cost type** | **ED only** | **CBE-led**  **Total 15-year** | **MG-led**  **Total 15-year** |
| --- | --- | --- | --- |
| **Health systems strengthening** | 97∙2 | 175∙9 | 189∙3 |
| **Direct treatment** | 107∙0 | 157∙0 | 357∙1 |
| Screening | 0∙0 | 15∙7 | 187∙6 |
| Diagnosis | 14∙5 | 33∙2 | 50∙8 |
| Treatment | 68∙4 | 81∙5 | 90∙8 |
| Palliative | 24∙1 | 26∙7 | 27∙8 |
| **Total** | **204∙2** | **232∙9** | **546∙4** |

10,300 13,500 15,500 (CBE); 16,800 (MG)


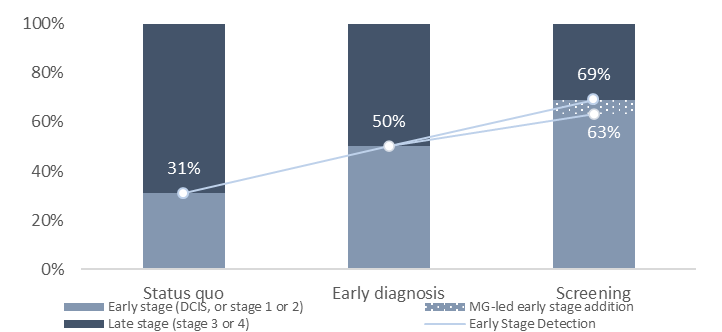


CBE-led scenario

MG-led scenario

**Costs:** Annual expenditures are a 0∙8, 1∙5, and 2∙5% increase over existing levels of GHE in scenarios 1-2b. Screening represents 39% of total cost in the MG-led scenario compared to only six percent in the CBE-led scenario.

**Diagnosis**: By 2037, with screening programs fully scaled, a higher share of women is diagnosed in early stages (63% versus 69% in the CBE-led or MG-led scenario).

**Treatment:** The *total* cost of treating increases because more breast cancer cases are diagnosed and treated. But average per-person treatment costs decrease as more early-stage cases are identified that require less intensive forms of therapy.

**Mortality reduction:** Survival rates improve as late-stage diagnosis decreases and more women receive treatment in early stages. Over 15-years, the ED , CBE-led, and MG-led scenarios respectively prevent 33,600, 44,900, and 50,600 deaths.

**15-year BCR**: ED scenario (1∙2); CBE-led screening (0∙8); MG-led screening (0∙6)

**Treatment – Average breast cancer treatment cost (USD) per case**

**Medium-term outcomes**

**2.3 Phase 3 (2038 to 2061) –** A mature breast cancer screening program in place

**What’s happening?** – A mature, biennial population-level screening program operates with women age 40 to 74 regularly screened for breast cancer. Strong referral structures facilitate diagnostic follow-up in women with abnormal findings, with linkages in place to ensure timely treatment for confirmed cases.

**Diagnosis – Global comparison of mammography machine capacity**

**40-year program & service delivery costs, USD millions (discounted)**


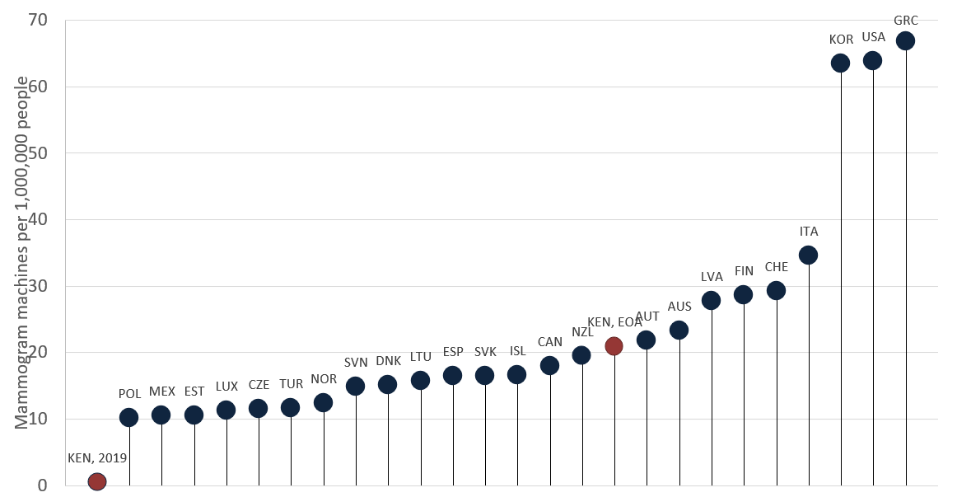


| **Cost type** | **Early diagnosis only** | **CBE-led**  **Total 15-year** | **MG-led**  **Total 15-year** |
| --- | --- | --- | --- |
| **Health systems strengthening** | 276∙9 | 589∙8 | 643∙0 |
| **Direct treatment** | 268∙1 | 474∙4 | 1,279∙1 |
| Screening | 0∙0 | 62∙3 | 745∙8 |
| Diagnosis | 33∙2 | 114∙8 | 190∙6 |
| Treatment | 166∙7 | 219∙1 | 257∙6 |
| Palliative | 68∙1 | 78∙2 | 85∙1 |
| **Total** | 545∙0 | 1,064∙2 | 1,922∙2 |

**Global data** – total # of dedicated MG machines in each country in 2019 (40). **Kenya projection** – minimum number of mammography machines required in MG-led scenario by end of analysis (EOA).

**Phase 1 Phase 2 Phase 3**

**Costs:** Respectively, annual expenditures are a 1∙4, 2∙8, and 5∙2% increase over current GHE in the ED, and CBE- and MG-led screening scenarios.

**Diagnosis**: The MG-led scenario requires at least 1,000 mammography machines operating in country by 2061, a number that would compare to current machine density among developed countries (see figure). CBE-led screening requires at least 60 machines, but more may be needed to ensure relative availability across regions.

**Treatment - Mortality reduction:** All scenarios significantly bend the projected mortality curve. Over the 40-year period, the ED scenario prevents over 163,000 deaths due to BC, the CBE-led scenario 236,000, and the MG-led scenario 270,000.

**40-year BCR:** In the ED scenario, benefits of the strategy begin to outweigh costs (break-even point) in 2035, and the BCR reaches 4.4 by analysis end (net benefits USD 1,833 million). CBE-led screening breaks even in 2040, with a BCR of 3.2 by analysis end (net benefits USD 2,298 million). MG-led screening breaks even in 2047, with a 2.0 BCR by analysis end (net benefits USD 1,910 million).

**Long-term outcomes**

**Breast cancer attributable deaths averted, by scenario**

**Treatment outcomes – number of lives saved, by scenario**
